# Effects of prostaglandin E1 on callus formation in rabbits

**DOI:** 10.1186/s12891-015-0695-y

**Published:** 2015-09-10

**Authors:** Pawel V. Lipinsky, Ivan V. Sirotin, Alexandr V. Skoroglyadov, Alexey V. Ivkov, Alexandr P. Oettinger, Evgeny E. Krynetskiy, Alexandr B. But-Gusaim, Andreas J. Roth

**Affiliations:** Traumatology, Orthopaedics Department, 64 Clinical City Hospital, Russian State Medical University, Vavilova 61, 117292 Moscow, Russia; Joint Implant Surgeons of Florida 7331 College Pkwy, Suite #300, Fort Myers, FL 33907 USA; Division for Total Joint Replacement/Orthopedics, Department of Orthopedics, University Hospital Leipzig, Traumatology and Plastic Surgery, Liebig Street 20, 04103 Leipzig, Germany

## Abstract

**Background:**

Recent research has focused on identifying chemical modulators of osteogenesis. We present initial findings on the osteoinductive properties of prostaglandin Е1 (Vasaprostan), using a rabbit model.

**Methods:**

Data were collected on callus formation in 14 male rabbits. These were divided into two groups (control and treatment) with 7 animals in each group. In all animals, the right tibia was fractured using a standardized protocol and stabilized by an intramedullary nail. Treatment group received a 5 μg/kg subcutaneous injection of PGE1/day during 10 postoperative days. Visual and radiological evaluation of callus formation was prospectively collected. After 30 days, all animals were killed and the tibia specimens were examined histologically.

**Results:**

In all the treatment group animals, fractures were consolidated radiologically by day 30. No treatment group animals and two control group animals were excluded form the experiment. In the control group, 4 animals demonstrated slower callus formation than the main group. Two control group animals were excluded from the experiment on the 20th day due to wound infection; one developed a nonunion.

The mean coefficient of bone callus thickening in the main group was 2.08 (±0, 16) and 1.77 (±0.05) (*p* < 0.05) in the control group. Calculation of mean quantity of neogenic vessels in 10 random visual fields of the bone callus revealed 78 (±9.82) in the main group and 40 (±4.68) in the control group (*p* < 0.05).

**Conclusions:**

Our study demonstrates an increased rate and amount of bone callus formation in the group treated with prostaglandin E1 compared to the control group. Prospective radiological analysis was corroborated by histologic evaluation.

## Background

Prostaglandin E1 (PGE1) has general vasodilatory, antithrombotic and endothelium-stabilizing properties. This is thought to be due to the activation of neutrophil leukocytes and thrombocytes, inhibition of the aggregation of erythrocytes and an increase of the elastic properties of erythrocyte cell walls. Furthermore, PGE1 shows fibrinolytic activity and was found to increase aerobic tissue respiration. Some literature describes the successful use of PGE1 in microsurgery for the replantation of extremities after traumatic amputation [1] and in patients after transplantation of musculo-cutaneous grafts [2]. Experimental work on prostaglandins *in vitro* indicated that prostaglandins of the E series had the greatest activity in bones [3–5]. It was reported in the early 1980s, that the infusion of PGE1 in human infants with congenital cyanotic heart disease led to bone proliferation on the periosteal surface of long bones after 3–4 weeks [6–8]. Infants that were given PGE1-infusions to prevent closure of the ductus arteriosus, developed prominent periosteal new bone formation. This finding was observed in radiological studies. Subsequently it was noted that this novel bone became incorporated into the cortex of the growing appendicular skeleton when PGE1 infusion was stopped [6]. In other early studies of the cardiovascular effects of prostaglandins, it was observed that intravenous infusion of PGE1 (25–250 μg/kg/min for 30 days) led to limb edema and periosteal and endocortical bone proliferation in dogs [9]. In mice, an increased endosteal bone formation has been found in long-term high dose studies of PGE1 analogues (misoprostol) [10]. Continuous local infusion of PGE1 (0.5–2.0 mg/week for 3 weeks) via sublingual cannula onto the surface of canine mandibles showed a stimulation of bone proliferation [11]. Both PGE1 and PGE2 locally injected into the fractured ribs of beagles (0.2 mg twice a day for 10 days) led to a greater proliferation and a surface area increase of osteoid on the surface of the fractured and contralateral non-fractured ribs when compared to a control group. This finding was more significant in the PGE1-group [12, 13]. PGE1 injected directly into a tibial cortical defect for the first 10 days led to a decreased proportion of bone in the callus, but a greater amount of periosteal new bone formation adjacent to the site [14]. From these results, it was concluded that PGE1 promotes differentiation as well as proliferation of osteoblasts *in vivo*. Although healing is not uniformly enhanced, exogenously administered prostaglandins have been found to enhance periosteal callus formation [14].

A comparative dose–response relationship of the effect of PGE1 on periosteal and intracortical bone in adult dogs (2–5 years old), infused by an osmotic mini-pump (0.0 to 16.7 mg PGE1/week) to the lateral mandibular surface or by controlled-release pellet (0.0 to 16.7 mg PGE1/week) was determined in a further study [15]. The proliferation of new bone at sites treated with the pellets was greatest with the 8.3 mg PGE1/week/three week treatment. New bone formation was observed, consisting of woven bone - particularly at sites with high accretion rates – as well as primary lamellar bone. Increases in the bone formation rates suggest that PGE1 increased the recruitment of osteoblasts. Increases in the mineral appositional rate, observed at a higher dose, seem to indicate that PGE1 stimulates bone production at the cellular level [15].

The synthetic analogue of PGE1 – Alprostadil – is used medically for obliterating atherosclerosis in vessels of the lower extremity. Based on the cited data, the authors suggested a possible positive effect of PGE1 in bone callus formation after fractures. To confirm this hypothesis, the authors performed a pilot study with an animal model in order to clarify PGE1’s mechanisms of action.

To answer the question whether PGE1 influences the fracture healing it was necessary to determine whether prostaglandins have an effect on the reparative processes in the bone tissue of the main group, treated with PGE1.

## Methods

The study was approved by a vote of the local ethics committee of the Pirogov’s Russian National Research Medical University of Moscow (Protocol №82; 15.12.2008).

For our experiment, we selected a model of minimally-invasive fracture of the tibia diaphysis with subsequent intramedullary osteosynthesis in rabbits as recommended by Sachno [16]. We chose this model, because the structure of a rabbit tibia allows for completely reproducible fracturing and permits suitable osteosynthesis. In all animals, the tibia was fractured transversally. Fibular fractures and small tibial bone fragment had no clinical value. As experimental animals, male rabbits were selected (age 2–3 years, weight 3–5 kg).

The animals were distributed into two groups with 7 animals in each group. In the control group, the tibia fracture was stabilized using intramedullary osteosynthesis, and no further treatment was carried out. In the main group, after fracturing the tibia and subsequent intramedullary osteosynthesis, treatment with PGE1 was performed.

Surgical procedures to create fractures and to stabilize them were carried out under anesthesia (Rometar 20 mg/ml (2 %), 0.1 mg/kg Zoletil 10 mg/kg subcutaneous). Bone diaphysis was exposed through a 1.5 cm skin incision in the anterior surface of middle third of the posterior right extremity. Then a 2 mm reamer was used to create three transversal canals with an angle of 90° to the bone diaphysis. Next, the bone was manually fractured at this level. For the intramedullary osteosynthesis two Bogdanov’s nails (ООО “ОСТЕОСИНТЕЗ”, Russia, Jaroslavskaja Oblast, Gorod Ruibinsk) were used. The transverse diameter of these Bogdanov’s nails was 1.2 × 2.4 mm; the length of the nails was determined intraoperatively and was 4 – 5 cm. For introduction of the nails in the intramedullary canal a supplementary 1.5 cm skin incision was made in projection of the patellar ligament. Distal to the ligamentous insertion, the intramedullary canal was opened with an awl. Then the two Bogdanov’s nails were introduced. The length of the nails was chosen so that they protruded approximately 1.5 to 2 mm from the bone in order to facilitate subsequent removal. After hemostasis was achieved, the wounds were closed. Drains and bandages were not used.

The control group of animals received only analgesic therapy during three postoperative days (Ketoprophenum 3 mg/kg body weight).

In addition to analgesic therapy, the main group of animals received a 5 μg/kg body weight subcutaneous injection of PGE1 (Vasaprostan) per day during 10 postoperative days at the contralateral thigh. The dosage and duration corresponds to other similar animal experiments [9, 11].

The state of the health of animals was examined during the entire postoperative period. Also the status of the operated limb (visually and manually free motion in knee and ankle joints, limping or not) and the postoperative wound conditions (visually healing, edema of soft tissues) were estimated.

The primary endpoint of the study was the callus formation, radiologically assessed at defined days, and histologically assessed at day 30.

The radiological examination of operated limbs (ap. and lateral view) was performed in the supine position on days 10, 20 and 30 following the operation. On the X-rays, the size, homogeneity, density, dynamics of bone callus formation, position of the bone fragments and the position of intramedullary nails were visually estimated.

On the 30th postoperative day, all animals were killed in accordance with the humane practice policy of our institution.

Tibia specimens with their callus were explanted and released from soft tissues. Intramedullary nails were removed. The bone was used for histological analysis by cross sections. These were fixed in paraffin and cut into10 μm layers. Samples were transferred onto microscopic slides and stained using hematoxylin, eosin and Van-Gieson’s methods. A conventional light microscope was used to examine the preparations.

To quantify the callus formation in relation to the bone cortex coefficient between the maximum diameter of bone with callus and the cortical diameter of native bone in cross sections of the tibia bone callus was recorded. Since the radiological examination could be influenced by inter- and intra-observer variability, a further pathomorphological study was performed on the decalcified slices. Maximum attention was paid to processes of neoangiogenesis and chondrocytogenesis. The quantity of neogenic vessels within the callus of each slice was counted in 10 random vision fields of light microscopy with tenfold magnification. Furthermore, the average number of vessels was calculated. The average number of chondrocytes was counted by the same method, but on 40-fold magnification.

The reliability of the experiment’s results was estimated by the non-parametric Wilkinson’s test for depended variables and by the Mann–Whitney test for independent variables. Statistica 7.0 software was used for performing the tests. The critical value of statistical error of the first kind was a p-value of 0.05 and less.

## Results

### State of health

The animals primarily showed signs of slight limping. Postoperatively, the weight-bearing ability of the extremity and the range of active motion in nearby joints were not significantly altered visually and completely restored after 10–15 days in the main group and after 20–25 days in the control group.

There were no complications during the postoperative period in the rabbits of the main group. Two animals of control group had a severe purulent inflammation of the soft tissue, so they were excluded from the experiment on the 20th day.

### Radiological examination

**Fig. 1:**
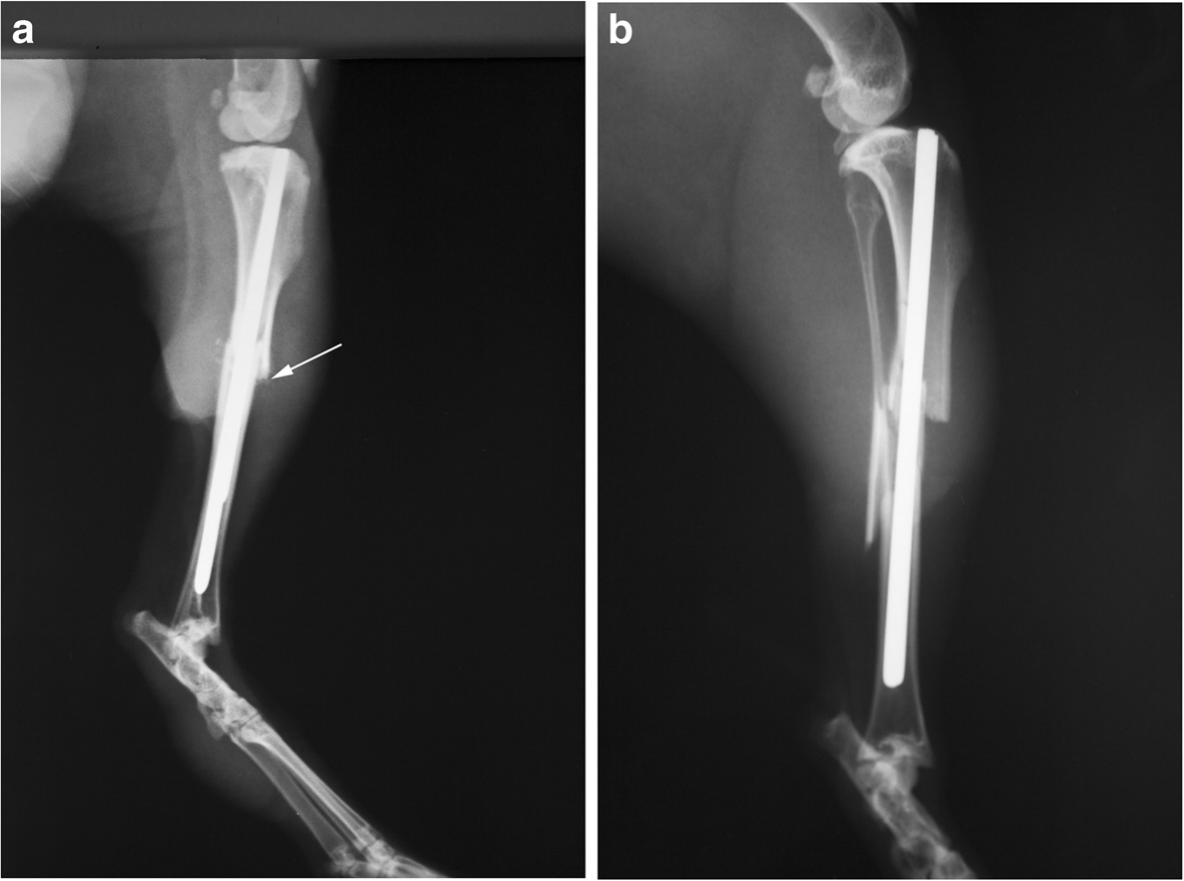
X-ray of the tibia on the 10th day in the main **(a)** and control **(b)** groups. There is more callus formation in group **a** The arrow point to bone callus

On the 10th day, all animals of the main group showed signs of bone callus formation on x-ray examination (Fig. 1), whereas the animals in the control group showed no such signs.

**Fig. 2:**
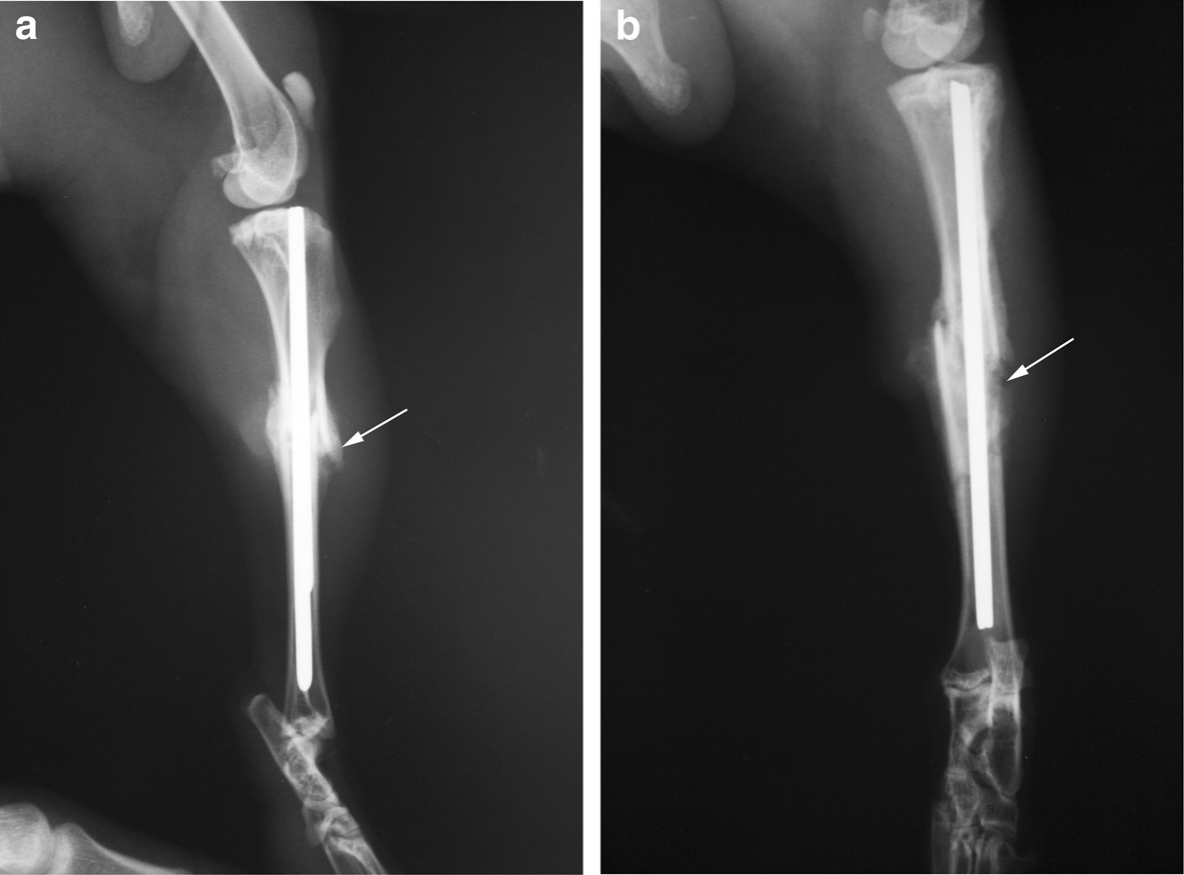
X-ray on the 20th day in the main **(a)** and control **(b)** groups. The callus formation in group **a** had progressed. In group **b**, 4 animals were found to have the same callus formation as in group A after 10 days. The arrows point to bone callus

On the 20th day, the same signs of bone callus formation, which were seen in the main group on day 10, appeared in 4 animals of the control group (Fig. 2). In 3 animals of the control group there were still no signs of bone callus formation at this stage. Two of the latter were excluded from the experiment on 20th day because of a wound infection.

**Fig. 3:**
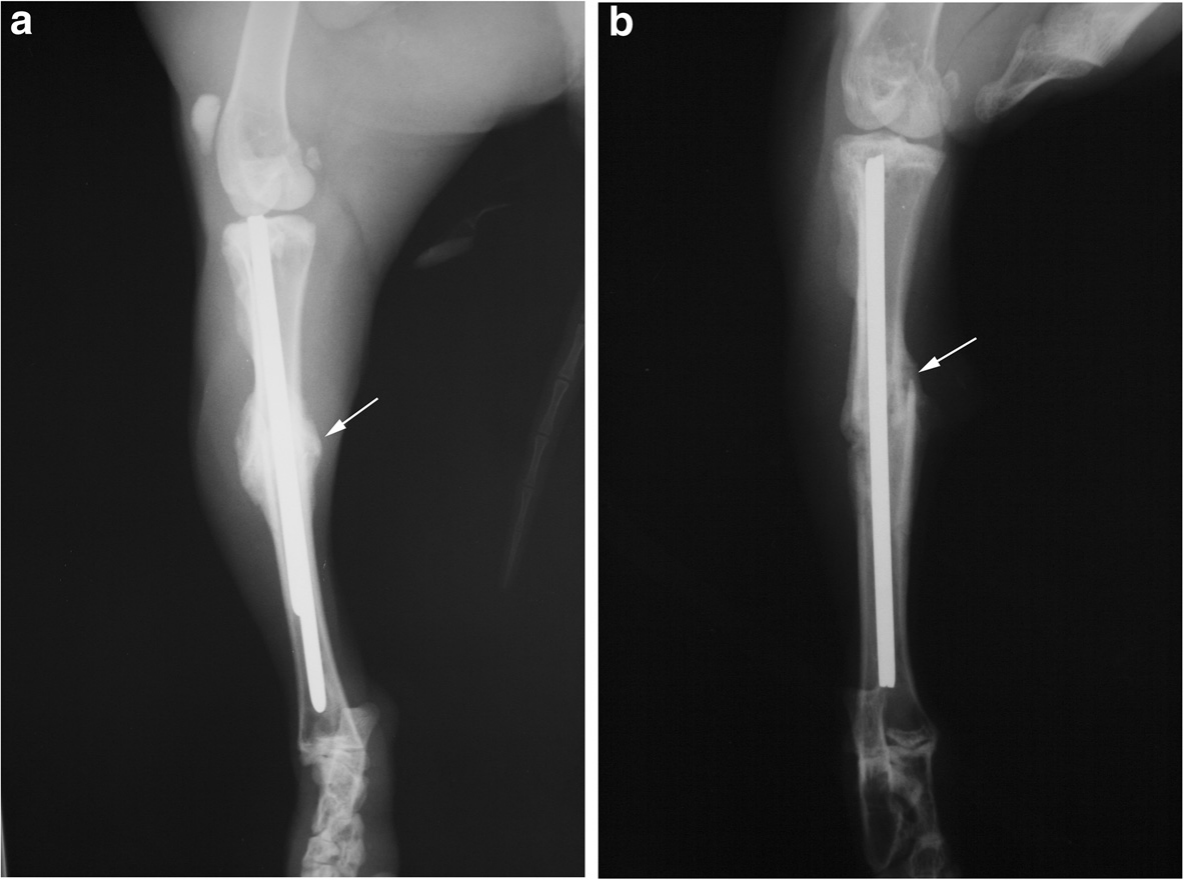
X-ray image on the 30-th day in the main **(a)** and control **(b)** groups. Fracture is completely consolidated in group a. In group **b**, the callus formation is comparable to group **a** after 20 days. The arrows point to bone callus

On the 30th day, in all animals of the main group, the fractures were radiologically consolidated. In the control group at this time, 4 animals showed similar callus formation as the main group on the 20th day (Fig. 3). On the 30th day, one animal in the control group had radiologically normal bone callus, but morphologically there was a non-union of fracture. In both groups, during the whole course, no excessive callus formation was observed.

### Histological examination

**Fig. 4:**
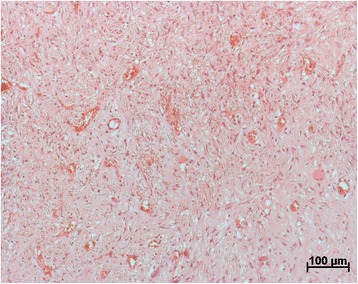
Neogenic vessels within the fibrous tissue at day 30 in the main group (HE-staining, 10x magnification). The calculation of mean quantity of the neogenic vessels in 10 random visual fields of the bone callus revealed with 78 (±9.82) in the main group a significant increased number compared to 40 (±4.68) in the control (*p* < 0.05)

On the 30th day it was morphometrically established that the mean coefficient of bone callus thickening in the main group was 2.08(±0, 16) and in the control group 1.77(±0.05). The difference of 18 %was significant (*p* < 0.05). The calculation of the mean quantity of neogenic vessels in 10 random visual fields of the bone callus, revealed 78(±9.82) in the main group and 40(±4.68) in the control group. This represented an almost twofold increase in the main group (*p* < 0.05) (Fig. 4).

**Fig. 5:**
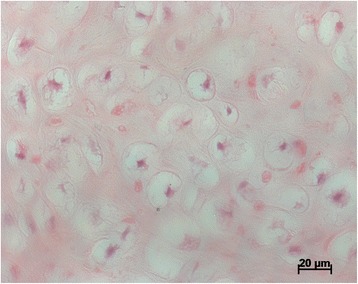
Chondrocytes in the main group at day 30 (HE-staining, 40x magnification). With 105 (±20.03) chondrocytes per 10 fields in the main group their number was significantly increased compared to 67 (±6.25) in the control (*p* < 0.05)

On the 30th day, the calculation of mean quantity of chondrocytes revealed 105(±20.03) chondrocytes per 10 fields in the main group and 67(±6.25) in the control group. This represented almost 150 % in the main group (*p* < 0.05) (Fig. 5).

## Discussion

The experimental model used in the presented study has been proven to be suitable to investigate the influence of PGE1 on fracture healing by its systematic use. The study has shown that the use of PGE1 in the postoperative period in the main group resulted in a decreased time to bone consolidation, represented by a faster rate of bone callus formation. Furthermore, in the control group, three animals showed no callus formation at day 20. It must, however, be noted that two of these animals were excluded from the experiment. At day 30 one animal in the control group still showed no callus formation. In contrast to this, all animals in the main group showed callus formation during the whole experiment.

These results confirm reports of other authors that PGE1 has a positive effect on reparative osteogenesis. Ozturk et al. [17] wrote about the positive effect of PGE2 in treatment of segmental radial bone defects in rats. They found a significant healing response in groups locally treated with PGE2 compared to other animals, not treated with PGE2 [17]. Li et al. [18] reported that the EP4 receptor - one of the subtypes of the PGE2 receptor - is a positive regulator in the maintenance of bone mass and fracture healing [18]. These results already pointed to an effect of prostaglandins in the context of fracture healing. Hommann et al. [19] used PGE1 systemically in patients after liver transplantation, because it is known that the majority of liver transplant patients develop bone mineral density (BMD) loss in the first 3 to 6 months post transplantation, which leads to an increased fracture risk. According to the data of the densitometry at the lumbar spine and the femoral neck, the authors found a reliable decrease of bone mass loss in patients who were not given PGE1. Furthermore, in the PGE1-treated main group, there was a significantly lower fracture rate compared with the control group [19]. Shih and Norrdin [12] have observed the positive effect after oral administration of PGE1 on regional haversian remodeling in beagles with fractured ribs and concluded that PGE1 can activate and synchronize remodeling cycles among BMUs [20]. All of these works suggest that PGE1 has an effect on bone formation. Sherbavskaja (2003) assumed PGE1 as a possible stimulator of bone tissue formation [21]. Similar data were described in studies of Riggs and Melton [22] and Marie [23], who could show a stimulation of the collagen synthesis and the secretion of insulin-like growth factor 1 (IGF-1) by PGE1. These authors concluded, that PGE1 has anabolic properties on bone tissue and stimulates bone metabolism.

The histological results of this study confirmed the radiological findings that there was an increased callus formation following treatment with PGE1. Thus, the mean coefficient of bone callus thickening was significantly increased in the main group. Furthermore an increased account of vessels and chondrocytes in the main group treated with PGE1 as a sign of increased proliferation of bone tissue was found. Although the increased amount of callus does not necessarily indicate a better fracture healing, it points to a local effect of PGE1 on the fractured bone. The work of Shih et al. [12, 13] showed that fractured ribs in the dog under the influence of PGE1 resulted in an increased formation of osteoid. Here, however, a local injection was used. PGE2 systemically given to dogs (6 mg/kg/day orally) during experimental rib fracture healing, led to a twofold increase in the amount of soft tissue callus at 2 weeks and a comparable increase in the amount of bony and cartilaginous callus at 6 weeks [24]. Systemic administration of PGE1 to improve fracture healing, as in the present work, has not been described in the literature.

## Conclusions

The obtained experimental data are convincing and demonstrate that in a setting of standardized experimental fracture with intramedullary fixation, the systemic use of PGE1 in the postoperative period with a dose of 5 μg/kg body weight per day, resulted in a decreased time period of bone callus formation when compared to stabilized, but additionally untreated fracture. Nonunion did not occur. Furthermore, in the main group, the mass of newly built callus was greater and the quality of the bone callus can be assumed to be higher, represented by more vessel and chondrocyte formation. Thus, the present study updates the data and develops the available information about the influence of PGE1 on neoangiogenesis and stimulation of bone tissue reparation under systemic application. The animal model used can be the basis of further investigations. However, the data of influence in bone tissue reparations after fractures were received for the first time and must be confirmed using a larger number of animals and a placebo control group. For further investigations, a CT analysis and the histological differentiation and measurement of fibrous tissue, cartilage and newly formed bone using histomorphometric measurements as described from Parfitt et al. [25] will be necessary. Further biomechanical investigations are necessary to confirm the estimation that these fractures heal more rapidly [25]. Regarding the time period, future studies would have to consider that remodelling probably starts after more than 30 days. As the persistence of chondrocytes was interpreted by other authors as a sign of delayed bone healing after locally administered PGE1 into the mandible [26], a longer observing period should be used in future to show if bone really develops. In this experiment, some animals failed in fracture healing due to local infections. Therefore, in order to guarantee the statistical relevance, future studies should primarily include more animals in the investigation.

## Abbreviations

ap.: Anteroposterior
BMD: Bone mineral density
BMU: Basic multicellular unit
cm: Centimeter
CT: Computed tomography
IGF1: Insulin-like growth factor 1
kg: Kilogram
mg: Milligram
μg: Microgram
ml: Milliliter
mm: Millimeter
PGE1: Prostaglandin E1
PGE2: Prostaglandin E2
